# Fungal Chitin Synthases: Structure, Function, and Regulation

**DOI:** 10.3390/jof11110796

**Published:** 2025-11-07

**Authors:** Linda Brain, Mark Bleackley, Monika S. Doblin, Marilyn Anderson

**Affiliations:** 1 Department of Ecological, Plant and Animal Science, La Trobe Institute for Sustainable Agriculture & Food (LISAF), AgriBio, Bundoora, VIC 3086, Australia; l.brain@latrobe.edu.au (L.B.); m.doblin@latrobe.edu.au (M.S.D.); 2 Department of Biochemistry and Genetics, La Trobe Institute of Molecular Science, La Trobe University, Bundoora, VIC 3086, Australia

**Keywords:** fungal chitin synthases, CHS, fungal cell wall, polysaccharide, cell wall inhibitors

## Abstract

Chitin is an essential polysaccharide of the fungal cell wall, critical for structural integrity, cell division and, in pathogenic fungi, virulence. As chitin is absent in both plant and mammalian systems, chitin synthases are considered attractive targets for the specific control of fungal pathogens. Yet despite decades of research, structural information on chitin synthases was lacking and inhibitors have failed to gain approval in the clinic. Current inhibitors are also ineffective against major agricultural pathogens such as *Aspergillus* and *Fusarium* species, largely due to the presence of multiple chitin synthase isoforms in filamentous fungi and the cell wall compensatory response induced under stress. However, recent cryo-electron microscopy structures of Class I chitin synthases from yeasts *Saccharomyces cerevisiae* and *Candida albicans* and an oomycete chitin synthase have provided unprecedented insights into the structural and mechanistic properties of these large, transmembrane proteins. These studies revealed conserved, domain-swapped homodimer architectures, distinct substrate binding and catalytic pockets, and sophisticated intrinsic regulatory mechanisms. With these breakthroughs, this review summarises our current understanding of fungal chitin biosynthesis, the challenges that remain to fully biochemically characterise these enzymes, and considers how the new structural insights may guide the development of broad-spectrum antifungals.

## 1. Introduction

Chitin is an key polysaccharide of the fungal cell wall, contributing to structural integrity and morphogenesis and is indispensable in cell division and pathogenesis [1]. Because this polymer is absent from mammals and plants, the biosynthetic enzymes responsible for its synthesis, the chitin synthases, represent promising targets for selective antifungal control in both clinical and agricultural settings [2,3].

However, despite several decades of research into chitin biosynthesis, the development of effective fungal chitin synthesis inhibitors has faced several challenges. Among these are the functional redundancy of chitin synthase (CHS) isoforms, with overlapping roles complicating functional studies [4]. The fungal cell wall salvaging response, in which inhibition of one cell wall polysaccharide induces a compensatory increase in others, further impacts inhibitor efficacy and contributes to intrinsic resistance in some fungal pathogens [5,6,7]. CHSs are also large, multi-transmembrane proteins that are difficult to express and purify in quantities sufficient for detailed structural and biochemical analysis [8]. As a result, high-resolution structures of CHSs remained elusive until recently, limiting our understanding of the mechanism of action of the few existing CHS inhibitors and preventing rational structure-guided design of improved compounds. Together these factors have hindered clinical development of CHS inhibitors such as nikkomycins and polyoxins [9,10].

Recent technical advancements in cryo-electron microscopy (cryo-EM) have resulted in an exponential increase in the number of solved membrane protein structures over the last decade [11]. Indeed, high-resolution structures of CHSs from yeasts, *Saccharomyces cerevisiae* and *Candida albicans,* and an oomycete, *Phytophthora sojae,* were solved recently using this approach [12,13,14,15]. These breakthroughs have provided unprecedented insights into chitin synthase architecture and mechanism of action including conserved domain-swapped homodimer configurations, distinct substrate binding and catalytic pockets, and sophisticated intrinsic regulatory mechanisms such as swinging gate loops and lipid plugs that control access to the translocation channel.

Despite these advances, significant gaps remain. Structural information of the remaining CHS isoforms is lacking and no CHS structures from filamentous groups exist. Consequently, the molecular basis for isoform-specific inhibitor sensitivity is unclear.

This review provides a comprehensive and updated view of fungal chitin biosynthesis, with an emphasis on the recent structural advances and their mechanistic implications. While recent reviews have explored the fungal cell wall architecture more broadly [16,17], this review focuses specifically on the molecular and structural biology of CHSs, integrating cryo-EM insights with decades of biochemical and genetic studies. We discuss the classification of CHS isoforms, structure–function relationships, and complex regulation from transcription to enzyme trafficking and activation at the site of chitin synthesis. Finally, we address the challenges of CHS inhibitor development, including structural diversity, functional redundancy, and compensatory cell wall responses and highlight strategies for future structure-guided antifungal drug discovery.

## 2. Chitin Structure and Function

Chitin is a linear homopolymer of β-1,4-linked N-acetylglucosamine (GlcNAc), with a tensile strength exceeding bone and steel. This remarkable quality arises from the assembly of microfibrils, whereby nascent chitin chains align and are further strengthened through intermolecular hydrogen bonds [1].

The orientation of chitin chains in this secondary structure results in different crystalline forms with different physicochemical properties, historically termed α-, β-, and γ-chitin, although additional forms have been observed in fungal cell walls. In fungi, chitin is present in predominantly the α-form, which is the most stable of chitin microfibrils [18]. The linear chains in α-chitin are arranged in an antiparallel array. In the β-form, chitin chains are oriented in parallel, and γ-chitin has two parallel chains and one antiparallel chain, although the latter could be considered a combination of the α- and β- forms [19,20].

Chitin is the second most abundant polysaccharide in nature after cellulose and is found in a wide range of taxonomic groups; fungi, molluscs, arthropods such as insects, crustaceans and spiders, and several other invertebrates [21]. In animals, chitin mainly provides protective and supportive roles, reinforcing the exoskeleton of arthropods, lining the inner body cavities of insects, and constituting the hard internal structures of molluscs [21]. In almost all fungi chitin is an essential component of the cell wall, providing structural integrity and is indispensable in cell division and pathogenesis [1].

Generally, chitin and β-1,3-glucan form a basket-like scaffold in the inner portion of the cell wall, via intrachain hydrogen bonding [22]. Traditional models of the fungal cell wall depict a distinct inner layer of chitin microfibrils, a thicker overlay of β-1,3-glucan and a variable adornment of mannosylated cell wall proteins and other minor polysaccharides attached via β-1,6-glucan linkages to the outer cell wall [22].

**Figure 1:**
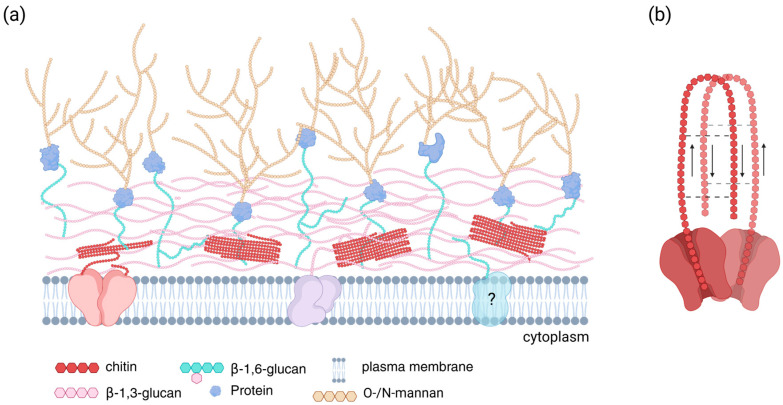
Generalised model of the fungal cell wall. (**a**) The inner layer of the cell wall is composed mainly of chitin microfibrils and β-1,3-glucan, synthesised by membrane-associated chitin synthases (pink) and β-1,3-glucan synthases (purple), respectively. The model proposed by Lenardon et al. [23] suggests that chitin is organised into bundles of microfibrils, providing flexibility for rapid expansion and adaptation. Cell wall proteins and other polysaccharides are covalently attached to the chitin-β-1,3 glucan scaffold via β-1,6- glucan cross-linkages. Mannosylated cell wall proteins are depicted in the outer layer of the cell wall, although the composition of this layer varies between fungal species [24]. The β-1,6-glucan synthase has been depicted as an integral membrane protein (blue), but its subcellular location remains uncertain [3] (marked by ? in the figure). (**b**) Chitin synthases are predicted to form dimers, and it is proposed that this higher-order organisation facilitates the pre-alignment of nascent chitin chains to form α-chitin microfibrils [12,13]. The figure is conceptually based on cell wall organisation models described by Gow and colleagues [16,17] and informed by recent structural and biochemical insights into CHS activity [13]. Created in BioRender by L. Brain. (https://BioRender.com/mrxjucn, accessed on 26 October 2025).

However, in recent years, this model has been reviewed and revised following detailed transmission electron microscopy and tomography of the *Candia albicans* cell wall [23]. The revised model considers the inherent inflexibility of chitin and the need for rapid adaptation and expansion of the cell wall during growth and osmotic stress. It is proposed that chitin microfibril bundles are interspersed throughout the lower, conserved layer of the cell wall rather than forming a discrete layer (Figure 1). In this arrangement, the rigid chitinous regions of the wall are complemented with the α-helical, branching structure of β-1,3-glucan that imparts strength and robustness but also the required elasticity [16].

The fungal cell wall is a dynamic structure. As the primary interface between the fungal cell and the environment, it is remodelled continuously in response to environmental pressures, developmental cues, and host immune attacks. Chitin is central to this process. Chitin deposition is spatially and temporally controlled by a complex regulatory network that coordinates the expression, transport, and activity of the enzymes responsible for its synthesis, CHSs [16,25,26].

## 3. The Chitin Biosynthesis Pathway

Common to all organisms capable of chitin synthesis is a conserved suite of biosynthetic machinery responsible for the conversion of sugars to N-acetylglucosamine to linear chitin chains [20]. This pathway can be divided into three distinct steps: the formation of N-acetylglucosamine (GlcNAc), its conversion to uridine diphosphate N-acetylglucosamine (UDP-GlcNAc), and chitin polymerisation. The first two sets of reactions take place in the cytoplasm whereas the last occurs at the plasma membrane primarily at budding sites in yeast and in the hyphal tips of filamentous species [1,20].

There are three highly regulated, critical enzymes in this pathway that determine the rate of chitin synthesis. The first committed step toward chitin biosynthesis is the formation of glucosamine-6-P from fructose-6-P and glutamine, a reaction catalysed by glutamine-fructose-6-phosphate aminotransferase (GFA1). The second critical reaction involves UDP-N-acetylglucosamine pyrophosphorylase (UAP1) which converts GlcNAc-1-P to UDP-GlcNAc [20]. Due to their importance in chitin biosynthesis, GFA1 and UAP1 are also being explored as potential antifungal drug targets [20,27,28]. The last reaction takes place at the plasma membrane by CHSs. CHSs utilise cytoplasmic UDP-GlcNAc as a substrate, catalysing multiple transfers of the sugar moiety to the non-reducing end of the growing chitin chain in a processive manner [20,29].

## 4. Fungal Chitin Synthases

CHS genes have been identified in almost all fungal species studied, as well as in bacteria, viruses, oomycetes, invertebrates, and some anamniote species [21,30,31,32,33,34]. However, with the exception of some insect and oomycete CHSs, functional annotation in many non-fungal species is lacking and the role or indeed the presence of chitin in these species has yet to be determined [35].

Fungi typically require multiple CHS isoforms for chitin biosynthesis, yeasts generally have one to three CHS genes, and filamentous fungi often have seven or more. Some filamentous species have more than 20 CHS-encoding genes in their genome [36,37]. The number of CHSs also typically correlates with the levels of cell wall chitin. For example, yeast cell walls can contain approximately 1–2% chitin, while filamentous species average around 15% [38]. However, there are many exceptions to this rule. Table 1 shows the number of CHSs, their predicted protein classes, and cell wall chitin content of some of relatively well-studied fungal species. Cell wall chitin in the dimorphic human pathogen, *Paracoccidioides brasiliensis* fluctuates dramatically between its yeast and hyphal forms. The yeast form has almost four-fold more chitin than its filamentous form, a feature shared with some other dimorphic species [39,40] although the inverse occurs with others [41]. *Fusarium* species typically have 12–14 predicted CHSs, one of the highest CHS counts within the Ascomycota division [37], though considerable variation in cell wall chitin content exists within the genus. For example, *Fusarium oxysporum* contains a relatively modest 8% cell wall chitin content while *Fusarium graminearum* can have up to 31% [42,43]. In summary, the biological functions of CHSs, particularly in filamentous and dimorphic species, appears to be species-specific and the necessity for multiple CHSs within certain groups remains a mystery.

### Classification of Fungal CHS Enzymes

**Figure 2:**
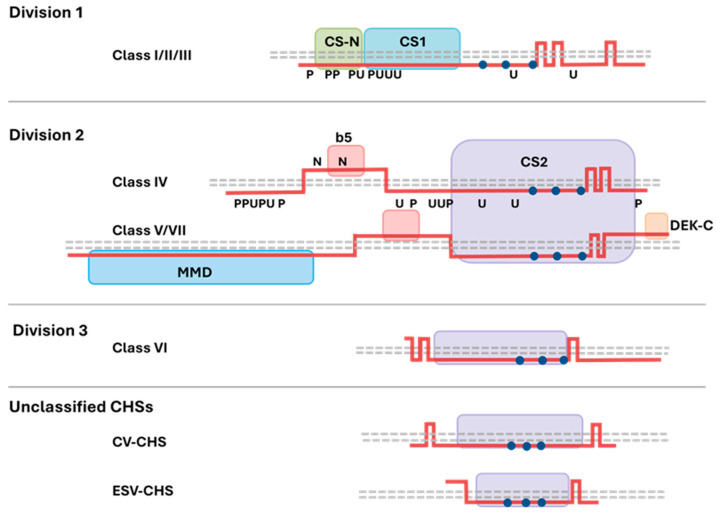
Predicted domain organisation of CHS proteins. Dashed grey lines represent the plasma membrane. The CHS proteins are shown as pink lines and vertical sections indicate transmembrane (TM) regions. Domains are coloured and the conserved motifs (QXXEY, EDRxL, and QRRRW) are indicated by blue circles. Predicted post-translational modifications (phosphorylation (P), ubiquiti-nation (U), and N-glycosylation (N) as determined by high throughput analysis of the *S. cerevisiae* proteome are labelled [87]. Division 1 (Classes I-III) CHSs contain a CHS N-terminal domain (CS-N), a CHS-1 domain (CS1), and a C-terminal multi-TM domain. Division 2 (Classes IV, V, and VII) CHSs contain a CHS-2 domain (CS2), a predicted extracellular region which may have a cytochrome b5-like heme/steroid binding domain (b5). Classes V and VII also exhibit a myosin head motor domain (MMD) and a DEK C-terminal domain (DEK-C). Division 3 (Class VI) and unclassified CHSs (CV-CHS and ESV-CHS) have a predicted CS2 catalytic domain, flanked by TM regions. The topology of Division 3 and unclassified CHSs are illustrated according to bioinformatic predictions by Goncalves et al. [37]. Division 1 (Classes I–III) and Division 2 (Classes IV, V, and VII) CHS architectures were adapted from Sánchez and Roncero [88] and expanded with additional CHS classes based on Gonçalves et al. [37]. Adapted and modified under the terms of the Creative Commons Attribution 4.0 International License (CC BY 4.0), https://creativecommons.org/licenses/by/4.0/.

CHS enzymes (EC 2.4.1.16) are members of the large glycosyltransferase 2 family (GT2) of processive β-glycosyltransferases. Glycosyltransferases are ultimately responsible for the biosynthesis of polysaccharides, oligosaccharides, and glycoconjugates, catalysing the formation of glycosidic bonds between the sugar residues of activated nucleotide sugar donors and specific acceptor molecules [44]. Other members of the GT2 family include hyaluronan and cellulose synthases, and although the GT2’s share a highly conserved catalytic domain, the members in this family have diverse substrate specificities [45,46,47]. The classic GT2 catalytic domain (GTD) includes the following motifs: DD, DxD, ED, and QXXRW, sequences of amino acids essential for substrate binding and catalytic function [45]. In CHSs, this cytoplasmic region includes the following sequences: QXXEY, EDRXL, and QRRRW, and is regarded as the CHS signature motif [48,49,50]. In addition to this region, CHSs are classified based on the presence of CHS like domains, which ultimately determine which division a CHS falls into. Figure 2 illustrates the typical predicted domain architecture of the different divisions of fungal CHSs.

Division 1 encompasses Class I, II, and III CHSs which have a CHS N-terminal domain (Pfam identifier: PF08407), a CHS-1 domain (PF01644), and a C-terminal transmembrane (TM) domain. Filamentous species possess all three classes of Division 1 CHSs, while yeast have only Class I and II. A gene duplication event has resulted in two Class III CHSs in some filamentous species, and subsequent sub-functionalisation arose as the two copies acquired different roles [36,37,51,52]. Division 1-like CHSs are present in fungi and oomycetes but not in animal genomes and are considered ancestral CHSs [53]. In oomycetes, the CHS N-terminal domain contains unique subdomains required for trafficking which are not found in fungal CHSs [54,55]. Division 2 CHSs include Classes IV, V, and VII which have a CHS-2 domain (PF03142) flanked by transmembrane regions [56,57]. Members of Division 2 often have a predicted cytochrome b5-like heme/steroid binding domain (PF00173), which is thought to have evolved in fungi to bind membrane-associated lipids [58]. Classes V and VII are only present in filamentous fungi and carry an N-terminal myosin head motor domain (PF00063) and a C-terminal DEK domain (PF08766). These two classes can be further distinguished by the length of the myosin head motor domain (MMD) and the presence of ATP-binding motifs [59,60]. The MMD has a role in trafficking and tethering the enzyme to the plasma membrane (PM) [61,62]. DEK proteins are associated with various chromatin-related processes in animals and plants [63,64], though the role of the DEK C-terminal domain in fungal Class V and VII CHSs is unclear.

Division 3 includes a Class VI CHS which is only found in some filamentous groups of the *Pezizomycotina* subdivision. Class VI CHSs contain a CHS-2 domain, flanked by transmembrane regions [37]. These more closely resemble non-fungal CHSs and other members of the GT2 family, such as hyaluronan and cellulose synthases, and are thought to have their own distinct ancestor [36]. The Class VI CHSs have evolved different roles in fungi. For example, in *B. cinerea*, the Class VI CHS is essential [65], but is dispensable for growth in *Sordaria macrospora* [66] and *Aspergillus fumigatus* [67]. It is also important for growth and development in *Neurospora crassa* [68] and appressorium formation in *Magnaporthe oryzae* [69].

Additional CHSs have been identified in filamentous fungi, and the classification of these proteins is uncertain. These include algae *Ectocarpus siliculosus* virus-like CHSs (ESV-CHS)s and chlorovirus-like CHSs (CV-CHS)s [37]. These smaller CHSs have domain structure similar to Class VI CHSs, with a CHS-2 domain flanked by TM regions and, depending on the study, fall into Division 2 or Division 3 [52]. It is proposed that these potentially ancient CHS sequences were transmitted to some fungal species from algal hosts or vice versa, via horizontal gene transfer [70]. Genes encoding enzymes which may provide CHS substrate precursors and chitin-modifying enzymes are often clustered with the viral-like CHSs and it has been suggested that this gene cluster was co-transmitted from the original host into fungi and may function as a unit [37,54]. ESV-CHSs are thought to be transcriptionally silent, although conversely CV-CHSs are differentially expressed during plant colonisation by the cereal pathogens, *Fusarium graminearum* and *Glomerella graminicola* [52]. One CV-CHS, termed *FgCHS8*, is important for normal cell wall development and pathogenicity in *F. graminearum* [71]. Interestingly, *Fusarium* species exhibit a propensity to accumulate these inter-kingdom CHSs more than any other fungal group studied [37].

**Table 1:** Comparison of the cell wall chitin content and the number and type of predicted CHSs across a diverse range of fungal species. CHSs are provided by their published names, while putative CHSs identified in large-scale genome studies [37,52] are presented with NCBI accession numbers and have not yet been verified experimentally.

			Division 1			Division 2			Division 3	Viral-like CHSs	
Species	Chitin %	Total CHSs	I	II	III	IV	V	VII	VI		Refs.
*Saccharomyces cerevisiae*	1–2%	3	Chs1	Chs2		Chs3					[56,72]
*Candida albicans*	2–6%	4	Chs2 Chs8	Chs1		Chs3					[73,74]
*Yarrowia lipolytica*	10%	7	Chs1	Chs2	Chs3	Chs4	Csm1	Csm2 Csm3			[75,76]
*Paracoccidioides brasiliensis*	40% (Y) 13% (H)	7	Chs1	Chs2	ChsB	Chs3	Chs5	Chs4	Chs6		[39,77]
*Ustilago maydis*	14% (Y) 16% (H)	8	Chs3 Chs4	Chs2	Chs1	Chs5 Chs7	Chs6 Chs8				[78,79]
*Aspergillus fumigatus*	7–15%	8	ChsA	ChsB	ChsC ChsG	ChsF	ChsE	CsmB	ChsD		[80,81]
*Blumeria graminis*	9% (C)	7	Chs1	Chs2	Chs3	Chs4	Chs5	Chs7	Chs6		[82]
*Neurospora crassa*	8%	7	Chs3	Chs2	Chs1	Chs4	Chs5	Chs7	Chs6		[68,83]
*Magnaporthe oryzae*	6–8%	7	Chs3	Chs2	Chs1	Chs4	Chs8	ChsV	ChsD		[69,84]
*Fusarium graminearum*	31%	13	Chs1	Chs2	Chs3a Chs3b	Chs4	Chs5	Chs7		Chs8 XP_011322595 XP_011325721 XP_011319624 XP_011321486	[43,71,85]
*Fusarium verticillioides*	28%	14	Chs1	Chs2	Chs3a Chs3b XP_018742086	Chs4	Chs5	Chs7	ChsD	XP_018754961 XP_018759373 XP_018750764 XP_018761653 XP_018757516	[86]

Chitin %: dry cell weight; Y: chitin in the yeast form; H: chitin in the hyphal form; C: chitin in conidia only.

## 5. CHS Structure and Mechanism of Action

The structure of CHSs remained elusive for decades, with early hypothetical models based on mutagenesis studies and parallels drawn with related processive GT2 enzymes. The crystal structure of a bacterial cellulose synthase complex from *Rhodobacter sphaeroides* provided the first structural insights into GT2 enzymes and a model for coupled synthesis and translocation of the polymer across the plasma membrane [89]. More recently cryo-EM structures of oligomeric forms of plant cellulose synthases from *Gossypium hirsutum* [90] and *Populus tremula × tremuloides* [91] and a viral hyaluronan synthase [92] revealed both conserved and unique features within the GT2 family enzymes.

Significant breakthroughs occurred in 2022 and 2023 with the first cryo-EM structures of CHSs, including Chs1 from oomycete, *P. sojae* [12], and Class I CHSs from the yeasts *S. cerevisiae* [14,15] and *C. albicans* [13]. These studies have deepened our understanding of the multistep mechanisms at play during chitin biosynthesis.

The structures revealed a conserved cytoplasmic glycosyltransferase domain (GTD), with a classical GT-A fold recognisable as a core of β-sheets surrounded by α-helices [93]. Each enzyme contains transmembrane domains (TMDs) consisting of six transmembrane helices, with three interfacial (IF) helices that tether the GTD at the cytoplasmic-PM boundary. This work corrected earlier topological models of CHSs, which had misidentified IF helices as TM helices [12,88].

**Figure 3:**
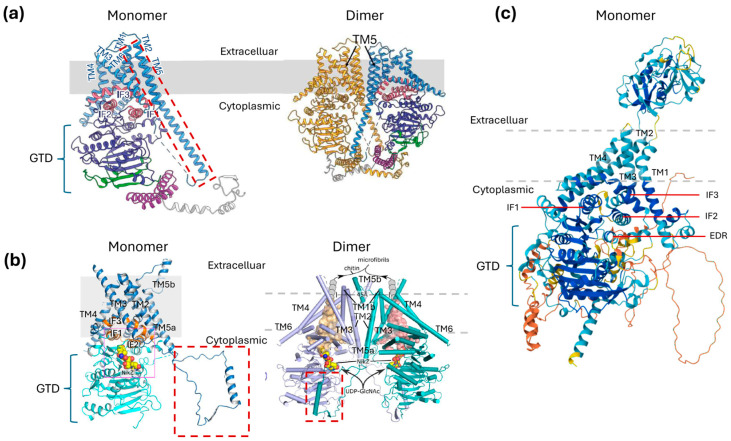
Structural comparison of oomycete and yeast CHSs. Cryo-EM structures of *P. sojae* PsChs1s (**a**) and *S. cerevisiae* ScChs1 (**b**) are shown in monomeric (**left**) and dimeric (**right**) forms [12,14]. These structures represent Division 1 CHSs. Each monomer comprises six transmembrane (TM) helices (TM1–6) forming the chitin translocation channel. The glycosyltransferase domain (GTD) adopts a canonical GT-A fold tethered to the TM region via three interfacial (IF) helices, shown in pink in (**a**) and orange in (**b**). Dimerisation of PsChs1 and ScChs1 involves TM5 and in ScChs1, a large cytoplasmic loop (regions are indicated by a dashed red box). The solved structure of *C. albicans* CaChs2 [13] closely resembles that of ScChs1. The plasma membrane (PM) is indicated with grey boxes or dashed lines with the extracellular region above and the cytoplasmic below. ScChs1 is shown with nikkomycin bound at the substrate binding site at the entrance to the chitin translocation channel (shown as yellow and orange space filling models on each protomer). Dimerisation is proposed to facilitate the formation of microfibrils (illustrated in ScChs1, right panel). Chitin polymers are depicted with grey hexagons. The AlphaFold model of ScChs3 (**c**) [100], a Class IV Division 2 CHS, reveals distinct structural differences compared with Class I enzymes. While the cytoplasmic region is similar to the Class I CHSs, ScChs3 possesses a unique extracellular N-terminal region connected by four TM helices (I–IV). IF helices 1 (QXXEY), 2, and 3 (QRRRW) are indicated along with the conserved EDR motif, which together form part of the catalytic core. Cryo-EM structures (**a**,**b**) were reproduced from Chen et al. [12] and Wu et al. [14], respectively, (licenced under CC BY 4.0) with additional annotations. The AlphaFold model of ScChs3 (**c**) was downloaded from the AlphaFold Protein Structure Database (DeepMind and EMBL-EBI; https://alphafold.ebi.ac.uk/entry/AF-P29465-F1, CC BY 4.0) with annotations added by the authors.

PsChs1, ScChs1, and CaChs2 have similar homodimeric structures (Figure 3). In PsChs1, dimer symmetry is centred on the long TM helix5 (TM5) extending from each protomer, forming a compact ‘snowflake’-like dimer (Figure 3a) [12]. The fungal Class I CHS dimer, however, has a more open configuration facing the cytoplasm, with dimerisation involving a shorter TM5 and a swap of a large cytoplasmic loop between the protomers (Figure 3b) [15]. The more closed configuration of the oomycete CHS dimer may represent an additional layer of control to substrate access, which could be related to the lower amounts of chitin present in oomycete cell walls compared with fungi [55]. Other fungal, insect, and oomycete CHSs are predicted to form higher-order structures [94,95,96]. Cellulose synthases, can also form multimeric complexes [45,97,98,99] and oligomerisation is considered a prerequisite for the correct assembly of microfibrils in vivo [20]. The spatially separated, parallel synthesis of individual chitin chains is predicted to facilitate microfibril assembly in the α-form [12,13].

The available high-resolution structures have thus far been limited to Class I CHSs [13,14,15], but AlphaFold modelling suggests that structural diversity exists within the CHS family [100]. For example, the AlphaFold model of ScChs3, a Class IV enzyme, shows a cytoplasmic catalytic region that resembles Class I CHSs, yet its overall architecture is markedly different (Figure 3c). Notably, ScChs3 harbours a distinctive N-terminal extracellular domain containing several N-glycosylation sites [101], positioning it for molecular interactions, which is connected to the catalytic core via four transmembrane helices. These unique structural features correlate with the higher levels of regulation in ScChs3 relative to Class I CHSs and will be discussed further in the context of ScChs3 trafficking in a later section.

### The Catalytic Mechanism for Chitin Biosynthesis

The cryo-EM structures have allowed an updated model for chitin biosynthesis. The GTD has distinct substrate binding and catalytic pockets for the biosynthetic reactions, while the TMD likely forms the chitin translocating channel (CTC). Access to the CTC is regulated in a CHS-specific manner. For instance, in the oomycete PsChs1 a swinging gate loop controls access to the CTC [12] and in the fungal ScChs1 the swinging gate loop is accompanied by an additional lipid plug which appears to control access to the channel and prevent non-specific leakage across the membrane [14,15]. This dual control over entrance into the translocation tunnel also occurs in a viral hyaluronan synthase [92]. The lateral lipid plug is also present in CaChs2, as well as an extracellular gate [13].

**Figure 4:**
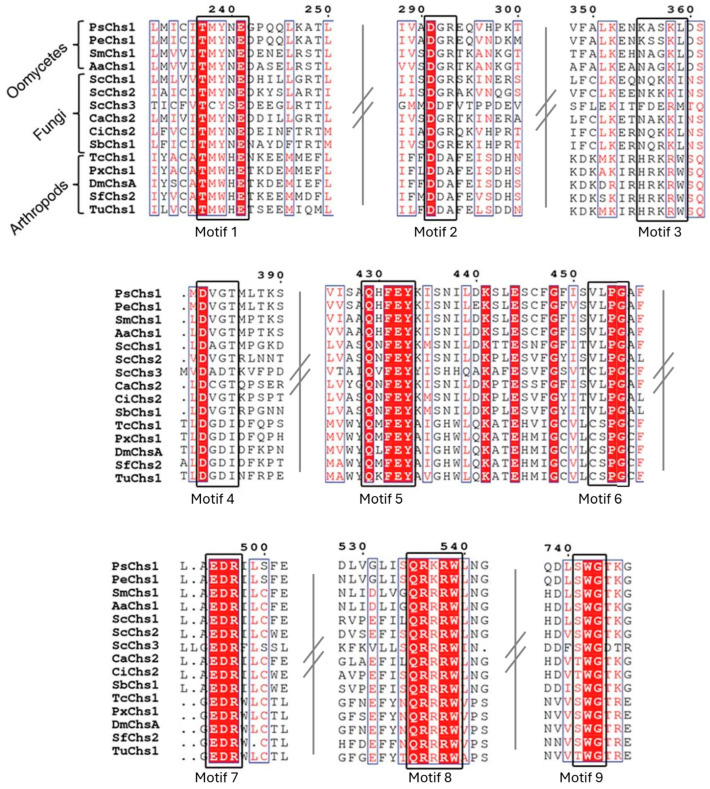
Conserved motifs in CHSs from oomycetes, fungi, and arthropods. The nine conserved motifs are marked with bold black boxes. Intervening sequences have been truncated for simplicity, indicated by a hashed line. Amino acids that are highly conserved are highlighted in white with a red background. Amino acids in red indicate similar residues and are marked in grey boxes and black amino acids indicate variable residues. Ps: *P. sojae*, Pe: *Peronospora effusa*, Sm: *Saprolegnia monoica*, Aa: *Aphanomyces astaci*, Sc: *S. cerevisiae*, Ca: *C. albicans*, Ci: *Coccidioides immitis*, Sb: *Sporothrix brasiliensis*, Tc: *Tribolium castaneum*, Px: *Plutella xylostella*, Dm: *Drosophila melanogaster*, Sf: *Spodoptera frugiperda*, Tu: *Tetranychus urticae*. Figure adapted from a multiple sequence alignment in Chen et al. [12] (licenced under CC BY 4.0), trimmed and reformatted to highlight motifs 1–9.

The Yamada-Okabe group originally described the central conserved catalytic region of CHSs, termed CON1 (including motifs QXFEY, EDR, QRRRW) and CON2 (WGTKG) [48,102]. This has now been extended to the series of nine conserved motifs illustrated in Figure 4, that are essential for substrate binding, catalysis, and translocation of the homopolymer into the cell wall space [12,13].

**Figure 5:**
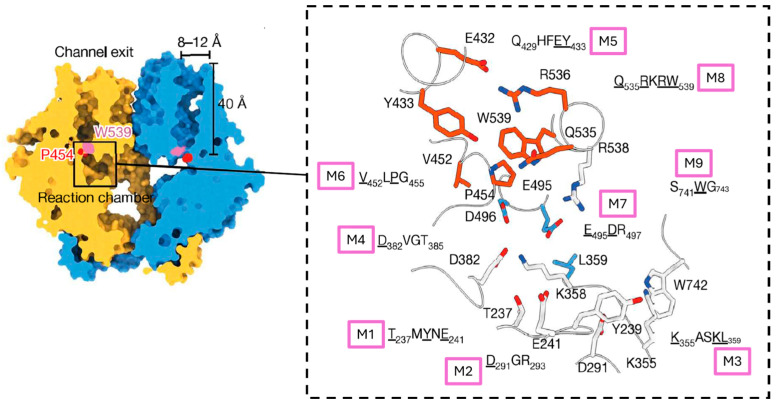
The reaction chamber of PsChs1. Sliced surface view of the PsChs1 dimer (**left**) showing the proposed chitin translocating channel (CTC). The reaction chamber is highlighted with a black box. The Pro454 (red) of the VLPGA motif and Trp539 (pink) of the QRRRW motif reside at the channel entrance. **Right**: Cartoon representation showing the conserved motifs of the reaction chamber. Residues important for activity are underlined and are shown as sticks. Critical residues forming the substrate binding pocket, catalytic cave, and CTC entrance are coloured grey, blue, and red, respectively. The substrate binding tub is formed by four motifs: TYMNE (M1), DGR (M2), KASKL (M3), and DVGT (M4), which are involved in nucleotide sugar binding. The ED motif (M7) resides within the catalytic cave where the aspartic acid facilitates transfer of the donor sugar. The CTC entrance includes motifs: QNFEY (M5), VLPGA (M6), and QRRRW (M8). The QNFEY and QRRRW motifs flank the channel entrance, positioning the substrate for catalysis, whereas VLPGA likely regulates access to the CTC and assists in polymer translocation. The SWG sequence (M9) may play a role in controlling substrate entry. The figure is adapted from Chen et al. [12] (licenced under CC BY 4.0), with motif annotations added by the authors.

Figure 5 depicts the active site of PsChs1 with the essential residues highlighted. The substrate binding tub is formed by motifs TYMNE (Motif 1), DGR (Motif 2), DVGT (Motif 4), and the KASKL motif (Motif 3) while substrate entry may be regulated by motif SWG (Motif 9, also known as CON2), located on a flexible cytoplasmic loop between IF3 and TM5, close to the reaction chamber [12].

Similarly to hyaluronan and cellulose biosynthesis, chitin synthesis is likely initiated by a self-priming mechanism [92,103]. Hydrolysis of UDP-GlcNAc occurs within the substrate binding tub, leaving monomeric GlcNAc positioned for elongation. The transfer of subsequent sugars involves the EDR motif (Motif 7) through an SN2-like displacement mechanism, in which the C4 hydroxyl group at the non-reducing end of the growing polymer attacks the anomeric carbon of the sugar moiety of the donor substrate, releasing UDP [104]. This process is also predicted for cellulose and hyaluronic acid synthesis [105]. The glutamate from the EDR motif binds the donor sugar to the substrate binding site and the catalytic aspartic acid serves as the general base that facilitates the nucleophilic attack. A divalent metal cation facilitates the release of the UDP moiety by binding the diphosphate of the leaving group and the β-1,4 glycosidic linkage between the two sugar residues is formed.

The entrance to the CTC includes motifs: QNFEY (Motif 5), VLPGA (Motif 6), and QRRRW (Motif 8). The QNFEY and QRRRW motifs reside on interfacial helices, IF1 and IF2, respectively. They flank the two sides of the channel and interact with each other via a salt bridge and hydrophobic interactions. The QRRRW motif may assist in the correct positioning of the substrate for catalysis. The third arginine of the QRRRW motif binds to the diphosphate of the donor substrate, while the tryptophan serves as a binding site for the acceptor substrate. The formation of the disaccharide likely induces a conformational change in the entrance to the CTC. The VLPGA motif is thought to serve as a gate lock to regulate access to the chitin translocation channel and it also stabilises the newly added sugar at the nascent end of the polymer. The flipping of the highly conserved proline of the VLPGA motif and possibly the displacement of the lipid plug in fungal CHSs is predicted to allow the nascent polymer to extrude through the channel into the cell wall space. GlcNAc residues are added in a processive manner, and it is believed that steric interactions could induce the rotation of the terminal sugar unit around the glycosidic bond and allow the sugar residues in the chain to be added in opposing orientations [98]. At completion of the chitin chain (on average 115–170 GlcNAc residues), the product is discharged, the enzyme reverts to a post-synthesis state, and catalysis may be initiated again [106].

This updated model for fungal chitin biosynthesis has revealed that fungal biosynthesis is intrinsically controlled at the enzyme level, ensuring that chitin chains are synthesised in the correct orientation and timing to support cell wall assembly and maintenance. In addition to these controls, CHS activity is highly regulated at a transcriptional and post-transcriptional level.

## 6. CHS Function and Regulation

Chitin biosynthesis has been studied primarily in yeast species and the roles for the individual CHS enzymes in yeast are well understood. *S. cerevisiae* has three CHSs: ScChs1, ScChs2, and ScChs3. ScChs1 and ScChs2 are involved primarily in cell division [107]. ScChs2 is essential, with roles in primary septum formation preceding division, and ScChs1 repairs the cell wall immediately after mother–daughter separation [108]. ScChs3 synthesises most of the lateral cell wall chitin and has a role in cell division by synthesising the chitin ring at the bud base [72,109]. The dimorphic yeast, *C. albicans* has four CHSs. CaChs1 (Class II) is essential and has overlapping roles with CaChs3 in septum formation and cell wall chitin synthesis (refer to Table 1) [73]. CaChs2 and CaChs8 (Class I) enzymes are not essential for survival but maintain cell wall integrity of yeast and hyphal cells during early polarised growth and during cell wall stress conditions [73,110].

Determining the function of CHSs in filamentous fungi is much more complicated than in yeast. The multiplicity of CHSs and discordant naming conventions have contributed to the ambiguity around anticipating orthologous CHS function in filamentous fungal species genomics [36,37,52,57,111]. CHSs in filamentous species exhibit overlapping roles and functional redundancy and show extreme diversity both between and within different taxonomic groups [37]. For instance, deletion of the Class III CHS in *F. graminearum*, *FgCHS3b*, was lethal, being important for growth and pathogenicity [51]. In contrast, in *A. fumigatus*, a double deletion mutant of the Class III CHSs, *AfCHSC* and *AfCHSG*, impaired growth but the fungus remained viable and was able to cause pulmonary disease in mice [112]. Double deletions of the Class V and VII CHSs within the *Aspergillus* genus caused lethality in *Aspergillus nidulans* [60] but not in *A. fumigatus.* In the latter, the double mutant had a disorganised cell wall structure, but the cells were viable, with normal levels of cell wall chitin [113]. As mentioned earlier, the roles of CHSs can be species-specific, and their function cannot be predicted based on purely on homology.

### Transcriptional Regulation

In yeast species, transcriptional changes to CHSs are mainly tied to the cell cycle and developmental stages. Transcripts of *ScCHS2* peak in M phase which coincides with its function in septum formation while *ScCHS1* peaks in G1 phase, corresponding with cell wall repair post-separation [114]. In contrast, *ScCHS3* is not transcriptionally regulated during the cell cycle but instead has complex post-translational controls [88]. In *C. albicans* CHS transcripts peak at G2 for all except *CaCHS2* (ortholog of *ScCHS1*), which is not cell cycle-regulated [115]. Differences in the transcriptional control of CHSs are attributed to the more complex life cycle of *C. albicans* as chitin synthesis is tightly regulated during yeast–hyphal transition [116]. Expression of CHSs in filamentous groups is less tied to the cell cycle as continuous chitin deposition is required at the hyphal tips for cell extension [115].

Transcriptional changes to CHSs also occur in response to cell wall trauma or cell wall perturbing agents via the activation of several cell wall salvaging pathways. These pathways include the cell wall integrity (CWI) pathway (also known as the protein kinase C or PKC pathway), the high osmolarity glycerol (HOG) pathway, and the calcium/calcineurin signalling pathway [115,117,118,119]. Cell wall disrupting agents such as Calcofluor White (CFW), Congo Red, caffeine, and β-1,3-glucan inhibitors can activate these pathways and induce a compensatory increase in cell wall chitin [118,120,121]. These signalling pathways and their role in fungal cell wall maintenance and antifungal tolerance have been comprehensively reviewed [119].

## 7. Post-Translational Regulation and Trafficking

All three of the *S. cerevisiae* CHSs are regulated at a post-translational level and have phosphorylated, cytoplasmic N-terminal regions ripe for protein modifications and interactions required for trafficking and function [101,122]. In yeast, transportation of CHS proteins to the PM involves interactions with multiple auxiliary proteins that ensure the delivery of the enzymes to the site where chitin synthesis occurs in a spatial and temporal manner [106]. CHSs lack an endoplasmic reticulum (ER) signal peptide sequence, and their synthesis, folding, and entry into the secretory pathway is unclear [88].

ScChs1 and ScChs2 are expressed as zymogens that require divalent metal cations, proteolytic activation, and oligomerisation for activity [14,122,123]. ScChs2 is cell cycle-regulated and short-lived. Dephosphorylation by Cdc14 at the end of mitosis allows the exit of ScChs2 from the ER and incorporation into COPII vesicles, after which it is transported to the septum site via the secretory pathway [87,124,125]. Association with Inn3 and Cyk3 at the division site activates ScChs2 by an unknown mechanism and septum formation ensues. At the completion of the septum, ScChs2 is ultimately inactivated via endocytosis and vacuolar proteolytic processing [106].

**Figure 6:**
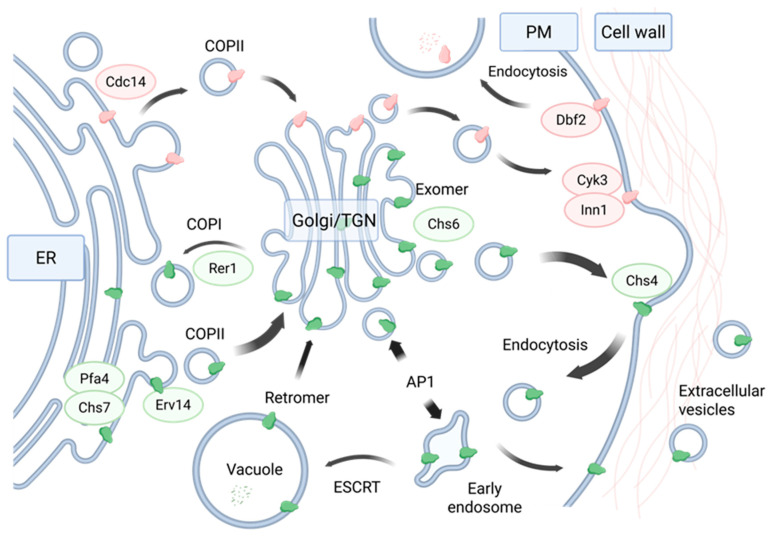
Trafficking of S. cerevisiae Chs2 and Chs3 enzymes. Proteins with known interactions with ScChs2 (pink icons) are shown in pink ovals and those with ScChs3 (green icons) are shown with green ovals. In ScChs3 trafficking, arrow width denotes relative trafficking volume. Top left: Dephosphorylation by Cdc14 allows exit from the endoplasmic reticulum (ER) at the end of mitosis and transport to the plasma membrane (PM) via the Golgi in COPII vesicles [87]. Once the mother–daughter cell junction is reached, Inn1 and Cyk3 activate ScChs2 by an unknown mechanism [106]. On completion of the primary septum, phosphorylation by Dbf2 mediates removal and subsequent degradation of ScChs2 in the vacuole [139]. Bottom right: ScChs3 exit from the ER depends on palmitoylation by Pfa4 and association with Chs7, which assists in the correct folding and subsequent recognition by the Erv14 cargo receptor for COPII loading [140]. The ScChs3–Chs7 interaction is maintained to the PM [126] but has been omitted for clarity. Sorting in the trans-Golgi network (TGN) via exomer subunit Chs6 allows transport to the PM [141]. Interaction with Chs4 activates ScChs3 and assists in anchoring it to the ring formation site [142]. At completion of chitin synthesis, ScChs3 is endocytosed and delivered to the early endosomal compartment where most of the protein is recycled back to the TGN in an AP1-dependent manner [143]. A small amount of ubiquitinated protein is delivered to the vacuole via the ESCRT complex for degradation, though some of this protein escapes degradation and is returned to the TGN via retromer [133]. Misfolded or non-oligomerised proteins are retrieved from the Golgi and shuttled back to the ER in a COPI and Rer1-dependent manner [101]. Some ScChs3 can reach the PM in the absence of AP1 or exomer via a poorly defined route [144]. Recycling of ScChs3 results in its accumulation within the TGN in intracellular vesicles termed chitosomes [124]. ScChs1 is also present in chitosomes [134], though its trafficking pathway is unclear and is not depicted. ScChs3 has been identified in extracellular vesicles (bottom right), with a proposed role in cell wall modification [135]. The schematic is compiled from Orlean [106] and Sánchez and Roncero [88]. Created in BioRender by L. Brain. (https://BioRender.com/qz1e0wo, accessed on 26 October 2025).

The trafficking of ScChs3 is more complex and has been recently reviewed by Sanchez and Roncero [88], who describe the roles of N-glycosylation, palmitoylation, phosphorylation, ubiquitination, and of auxiliary proteins involved in the delivery to the PM. These critical steps are summarised in Figure 6, alongside the trafficking of ScChs2.

Within the ER, N-glycosylation of the luminal N-terminus is thought to shield ScChs3 from ER-associated degradation (ERAD). Through its N-terminal domain, ScChs3 forms at least a dimer and an association with ScChs7 facilitates its exit from the ER via transport in COPII vesicles [101]. The precise interaction between ScChs3 and ScChs7 is unclear, although the C-terminal region of ScChs7 is involved, and the association is maintained along the secretory route to the PM [126]. In the *Saccharomyces* genus, the delivery of the ScChs3 complex to the PM relies on exomer, a *trans*-Golgi network sorting complex [127]. ScChs3 does not require proteolytic activation [128], but instead requires an interaction with ScChs4 at the synthesis site for docking at the PM and full activation [72,129]. In addition to facilitating delivery to the PM, ScChs4 probably interacts with the catalytic domain of ScChs3, enabling it to adopt a conformationally active form [130]. This association is likely to delay endocytic turnover and thus extends the residence time of ScChs3 at the PM, resulting in increased chitin synthesis at the site [131].

At the completion of synthesis, ScChs3 is retrieved from the PM in endosomal compartments and small amounts of ubiquitinated protein are trafficked to the vacuole for degradation [88]. However, the majority of ScChs3 is recycled, retrieved from the endosomal compartments by binding of the AP-1 complex at its N-terminal cytoplasmic domain, and re-enters the *trans*-Golgi network. This pool of inactive ScChs3 forms a major intracellular reservoir in vesicles termed chitosomes, ready for rapid mobilisation and re-activation [132,133]. ScChs1 has been detected in chitosomes along with ScChs3, suggesting that it may follow a similar recycling mechanism, although how it is trafficked to these vesicles remains unclear [134].

ScChs3 had also been found in the cargo of extracellular vesicles and a role for the intercellular delivery of CHSs in cell wall remodelling and resistance to antifungals has been proposed [135].

In summary, ScChs3 regulation is based mainly on post-translational modifications, the controlled rate of endocytic turnover and rapid deployment [88].

Post-translational regulation is also critical for the delivery and activation of CHSs of filamentous fungi to the extending hyphal tips. Class V and Class VII CHSs do not require the Chs7 chaperone for exit from the ER but are shuttled within intracellular vesicles to the synthesis site via interactions with the cytoskeleton through their MMDs [61,136]. Interestingly, the MMD also appear to facilitate the delivery of β-1,3-glucan synthases within the same vesicles, ensuring the co-synthesis of chitin and β-1,3-glucan at the hyphal tips [137,138].

## 8. CHS Inhibitors

The intrinsic regulatory mechanisms and complex post-translational control over fungal CHS activity presents both challenges and opportunities for CHS inhibitor development.

Benzoylureas, which are chitin synthesis inhibitors, have been used as insecticides for years, though they are ineffective against fungi. Their precise mechanism of action is unclear, though they are thought to act on a post-catalytic step in chitin synthesis, possibly by interfering with the translocation pore or the assembly of microfibrils. This may reflect structural differences between insect and fungal CHSs in this region [145].

Chitin synthase inhibitors that are active against fungi include polyoxins and nikkomycins, which are structural analogues of UDP-GlcNAc and are potent competitive inhibitors of certain fungal CHS isoforms [146,147,148].

Polyoxin D is used as an agricultural fungicide for the management of turf fungal blight and grey mould of strawberries (*Botrytis cinerea*); however, in vitro studies have shown that resistance to polyoxins can develop with repeated exposure [149,150,151]. Additionally, reduced susceptibility to polyoxin D has been observed in isolates of *B. cinerea* even in the absence of prior exposure suggesting the presence of inherent genetic resistance [151]. Natural resistance may also explain the limited efficacy of polyoxins against the growth *Aspergillus* and *Fusarium* species [6,7].

Nikkomycins have been under clinical development for decades [10] and show synergistic activity on fungal growth with azoles and echinocandins [6,152,153]. However, efficacy remains variable across fungal pathogens and, to date, no CHS inhibitor has been approved for clinical use [154].

Recent cryo-EM studies revealed that nikkomycin Z and polyoxin D bind within the substrate binding pocket of Class I CHSs, occupying the reaction chamber and thereby completely blocking the entrance of the substrate and preventing chain elongation [12,14]. In CaChs2 this interaction was more favourable with nikkomycin Z than UDP-GlcNAc itself [13]. Although both inhibitors block the transport channel, nikkomycin Z extends further into the translocation tunnel, whereas polyoxin D stops at the donor binding site, possibly explaining the stronger inhibitory effect of nikkomycin Z [13,155].

Despite the conservation of the critical motifs involved in inhibitor and substrate binding, inhibitor sensitivity varies between CHS isoforms. It is conceivable that variation in the intervening sequences surrounding the motifs alters the local environment of the binding pocket, influencing inhibitor binding affinity. Further, variation in the mechanisms for regulating enzyme access have already been observed within Class I CHSs [13,14,15], making it likely that the other CHS isozymes from different divisions also share this diversity. Structure determination of the remaining CHS classes is critical to reveal variations in the active sites of these enzymes, or unique mechanisms regulating enzyme access.

Adaptive cell wall responses further complicate effective inhibitor discovery. The core structure of chitin and β-1,3-glucan in the fungal cell wall is so important that cell wall stress signalling pathways have evolved to maintain cell wall integrity by inducing a compensatory increase in one when the other is diminished [119,156,157]. Simultaneous inhibition of both β-1,3-glucan and chitin biosynthesis represents an attractive strategy to bypass the cell wall adaptive stress response, provided multiple CHSs can be inhibited [3,158].

The availability of high-resolution structures of the Class I CHSs from yeasts offers new opportunities for antifungal discovery. Detailed architecture of the active site, CTC, intrinsic control mechanisms and regulatory regions provide a critical template for rational inhibitor design. Other attractive targets include the N-terminal regulatory regions of CHS enzymes, which mediate oligomerisation, trafficking, interactions with accessory proteins, and activation. The Class V and Class VII CHSs from filamentous fungi are also interesting targets because they are essential for virulence and are co-transported with β-1,3-glucan synthases [137,138], raising the possibility of dual control over the adaptive cell wall response.

Finally, AI-assisted inhibitor design offers an exciting avenue for the development of antifungals. With the recent structural information available, machine learning models can be applied to virtually screen chemical libraries for compounds that can bind to the CHS active site, regulatory regions or accessory protein interaction sites [159]. Further, AI-guided approaches coupled with medicinal chemistry could be used to engineer derivatives of nikkomycins and polyoxins with improved isoform coverage. Such strategies may enable the development of broad-spectrum CHS inhibitors, capable of overcoming the CHS isoform redundancy and the adaptive cell wall compensatory response. In summary, the integration of the recent structural insights and the prospect of AI-assisted inhibitor design make CHS inhibitor development a promising direction for antifungal drug discovery.

## 9. Conclusions and Future Directions

Fungal chitin synthases remain an underexploited target for the control of fungal pathogens. Despite over half a century of research into chitin biosynthesis, the development of effective CHS inhibitors has been hampered by the complexity of fungal cell wall biogenesis, the functional redundancy of CHS isoforms, and the lack of detailed mechanistic and structural information.

The recent cryo-EM structures of Class I CHSs from yeasts *S. cerevisiae* and *C. albicans* and the oomycete *P. sojae* represent a pivotal breakthrough in chitin biosynthesis research [12,13,14,15]. These advances have transformed our understanding from hypothetical homology-based models [89], to detailed molecular mechanisms of chitin synthesis. We can now describe how CHSs mediate substrate binding, catalysis, and chitin translocation governed by sophisticated intrinsic regulatory mechanisms, such as swinging gate loops, lipid plugs, and domain-swapped architectures [12,13,14,15]. Combined with decades of research on transcriptional regulation [4,118], post-translational modifications [87], and trafficking pathways [88,106], we now have a comprehensive view of how CHSs are regulated, from gene expression through to activation and catalysis.

However, the complexity of these processes also explains why fungal CHSs have been such challenging antifungal targets. The multiplicity of CHS isoforms [37], each with distinct architectures, regulatory elements [12,13,14,15], and trafficking pathways [88,106], creates functional redundancy that allows fungi to compensate when individual CHSs are inhibited [4]. While fungal CHSs share a conserved catalytic site, variations in the overall structures and regulation are likely to influence inhibitor efficacy and complicate the design of broad-spectrum inhibitors. Furthermore, CHSs exhibit species-specific functions, meaning CHS roles cannot be reliably inferred on sequence homology alone [4]. This problem is exacerbated in filamentous fungi, which possess additional classes (V, VI, VII) and in some cases harbour virally derived CHSs [37], making insights from model yeasts poor predictors of inhibitor binding and activity. Even within filamentous fungi, functional divergence of CHSs occurs. For example, FgChs3b, a Class III CHS, is essential in *F. graminearum* [51] but its counterpart in *A. fumigatus* is dispensable for growth [112]. Furthermore, the cell wall salvaging response, triggered when chitin synthesis is inhibited, induces a compensatory increase in β-1,3 glucan production, which undermines single-target strategies [156]. These challenges have prevented current fungal CHS inhibitors, nikkomycins and polyoxins, from entering the clinic [9], despite their potent competitive inhibition of Class I CHSs [12,13,14].

Significant gaps in our understanding of chitin biosynthesis remain. High-resolution structures are limited to the Class I fungal chitin synthases and no structures of the remaining CHS classes, or any CHSs from a filamentous species are yet available. While AlphaFold can predict a model for Division 2, Class IV CHSs [100], computational predictions cannot inform us of the dynamic regulatory mechanisms. Class V and VII CHSs are of particular interest for controlling filamentous fungal pathogens, because their N-terminal MMDs have been shown experimentally to facilitate vesicular trafficking of both CHSs and β-1, 3-glucan synthases to the hyphal tips [61,138], yet no computational or structural models currently exist for these critical enzymes. The variable activity of nikkomycins and polyoxins across different CHS isoforms suggests that important drug–enzyme interactions cannot be predicted from the Class I CHSs alone. Although the residues of the active site is conserved among fungal CHSs [37], differing overall architecture and regulatory elements likely explain the differential inhibitor sensitivity. While post-translational regulation and auxiliary protein interactions of yeast CHSs and are well characterised, they remain largely unexplored in filamentous fungi, where regulatory mechanisms are expected to differ, due to the requirement for continuous chitin deposition at the growing hyphal tips and to reinforce the cell wall during host invasion [160].

These gaps have defined clear research priorities. High-resolution structures of the remaining CHS classes, particularly those from filamentous pathogens, are essential to reveal class-specific variations that can guide structure-guided inhibitor design. Given what we know about the multi-tiered regulatory network governing CHS activation and synthesis, yeasts represent an ideal expression system, as these elements are already present. Indeed, Chen et al. [15] solved the cryo-EM structure of ScChs1 by heterologous expression in *S. cerevisiae*, suggesting that ScChs1 may be less tightly regulated in yeast than in ScChs2 and ScChs3. However, successful heterologous expression of other CHS classes may require circumnavigating the intrinsic mechanisms that control the overexpression of CHSs in yeast, such as post-translational modifications and degradation pathways that limit CHS accumulation [101].

Targeting the N-terminal region presents a promising strategy for broad-spectrum CHS inhibition as this region is critical for oligomerisation, trafficking, auxiliary protein interactions and activation in yeast CHSs of Class I, II, and IV. [87,88,101]. Further research is needed to determine if the N-terminal region of filamentous CHSs share these regulatory features, although sequence conservation suggests they may [37,52]. Notably, the N-terminal MMD of Class V and Class VII CHSs of filamentous fungi is also critical for trafficking of these enzymes to the hyphal tips [61], implying that despite their structural divergence, regulation through their N-terminal elements may be a shared feature of CHS isoforms. Furthermore, Class V and Class VII CHSs are of particular interest as inhibitor targets because they are essential for virulence and co-transported with β-1,3-glucan synthases [137,138], potentially bypassing the adaptive cell wall stress response with dual targeting.

The integration of structural biology, computational modelling and artificial intelligence has created unprecedented opportunities for the development of effective CHS inhibitors. The high-resolution structures can enable virtual screening and AI-assisted refinement of existing scaffolds such as nikkomycins, into more potent, broad-spectrum derivatives [155,159]. Computational methods could also identify novel targets within regulatory domains, such as auxiliary protein interaction sites.

The significance of CHS research extends beyond fundamental fungal biology to addressing real-world global health challenges. Invasive fungal infections pose an escalating threat to human health, especially among immunocompromised individuals. With limited treatments and antifungal resistance on the rise, new therapeutics are urgently needed [9]. Concurrently, fungal plant pathogens pose a significant threat to global food security, a problem further exacerbated with a changing climate [161]. Absent from plants and mammals, CHS enzymes represent one of the few fungal-specific targets available for antifungal development [2]. By integrating biological and structural insights with emerging computational strategies, the field is now poised to make significant progress in the development of next-generation antifungals but will require sustained research investment to deliver.

## Data Availability

No new data were created or analyzed in this study. Data sharing is not applicable to this article.
